# Food allergy and anaphylaxis – 2052. Vitamin D insufficiency is associated with challenge-proven food allergy in infants

**DOI:** 10.1186/1939-4551-6-S1-P135

**Published:** 2013-04-23

**Authors:** Katie Allen, Jennifer Koplin, Anne-Louise Ponsonby, Lyle Gurrin, Melissa Wake, Peter Vuillermin, Shyamali Dharmage

**Affiliations:** 1 Department of Paediatrics, University of Melbourne, Australia; 2 Murdoch Childrens Research Institute, Australia; 3 Centre for MEGA Epidemiology, University of Melbourne, Australia; 4 Centre for Community Child Health, Royal Children's Hospital, Australia; 5 Child Health Research Unit, Barwon Health and Deakin University, Australia

## Background

Epidemiological evidence has shown pediatric food allergy is more prevalent in regions further from the Equator, suggesting vitamin D insufficiency may play a role in this disease. We investigated the role of vitamin D status in infantile food allergy.

## Methods

A population sample of 5,276 one-year-old infants underwent skin prick testing to peanut, egg, sesame and cow’s milk/shellfish. All of those with a detectable wheal, and a random sample of skin prick test negative participants, attended a hospital-based food challenge clinic. Blood samples were available for 577 infants (344 with challenge-proven food allergy; 74 sensitizated but tolerant to food challenge; 159 negative both on skin prick and food challenge). Serum 25(OH) D levels were measured using liquid chromatography tandem mass spectrometry. Associations between serum 25(OH) D and food allergy were examined using multiple logistic regression, adjusting for potential risk and confounding factors.

## Results

Infants of Australian-born parents, but not of parents born overseas, with vitamin D insufficiency (<50 nM/L) were more likely to be peanut (aOR 12.22, 95% CI 2.55, 58.61, p=0.002) and/or egg (aOR 7.26, 95% CI 2.52, 20.91, p<0.001) allergic than those with adequate vitamin D levels. Those with vitamin D insufficiency were more likely to have multiple (≥ 2) than single food allergies (aOR 16.29, 95%CI 4.07, 65.27 vs aOR 2.72, 95%CI 0.45, 16.23 respectively) independent of eczema status.

## Conclusions

These results provide the first direct evidence that vitamin D sufficiency may be an important protective factor for food allergy in the first year of life.

